# Cost-Effectiveness of Early Detection and Prevention Strategies for Endometrial Cancer—A Systematic Review

**DOI:** 10.3390/cancers12071874

**Published:** 2020-07-11

**Authors:** Gaby Sroczynski, Artemisa Gogollari, Annette Conrads-Frank, Lára R. Hallsson, Nora Pashayan, Martin Widschwendter, Uwe Siebert

**Affiliations:** 1 Institute of Public Health, Medical Decision Making and Health Technology Assessment, Department of Public Health, Health Services Research and Health Technology Assessment, UMIT—University for Health Sciences, Medical Informatics and Technology, A-6060 Hall i.T., Austria; gaby.sroczynski@umit.at (G.S.); artemisa.gogollari@umit.at (A.G.); Annette.Conrads-Frank@umit.at (A.C.-F.); lara.hallsson@umit.at (L.R.H.); 2 Division of Health Technology Assessment and Bioinformatics, ONCOTYROL-Center for Personalized Cancer Medicine, A-6010 Innsbruck, Austria; 3 Department of Applied Health Research, University College London, London WC1E 6BS, UK; n.pashayan@ucl.ac.uk; 4 Department of Women’s Cancer, University College London, London WC1E 6BS, UK; m.widschwendter@ucl.ac.uk; 5 Center for Health Decision Science, Department of Health Policy and Management, Harvard T.H. Chan School of Public Health, Boston, MA 02115, USA; 6 Institute for Technology Assessment and Department of Radiology, Massachusetts General Hospital, Harvard Medical School, Boston, MA 02114, USA

**Keywords:** endometrial cancer, cost-effectiveness, prevention, decision analysis

## Abstract

Endometrial cancer is the most common female genital tract cancer in developed countries. We systematically reviewed the current health-economic evidence on early detection and prevention strategies for endometrial cancer based on a search in relevant databases (Medline/Embase/Cochrane Library/CRD/EconLit). Study characteristics and results including life-years gained (LYG), quality-adjusted life-years (QALY) gained, and incremental cost-effectiveness ratios (ICERs) were summarized in standardized evidence tables. Economic results were transformed into 2019 euros using standard conversion methods (GDP-PPP, CPI). Seven studies were included, evaluating (1) screening for endometrial cancer in women with different risk profiles, (2) risk-reducing interventions for women at increased or high risk for endometrial cancer, and (3) genetic testing for germline mutations followed by risk-reducing interventions for diagnosed mutation carriers. Compared to no screening, screening with transvaginal sonography (TVS), biomarker CA-125, and endometrial biopsy yielded an ICER of 43,600 EUR/LYG (95,800 EUR/QALY) in women with Lynch syndrome at high endometrial cancer risk. For women considering prophylactic surgery, surgery was more effective and less costly than screening. In obese women, prevention using Levonorgestrel as of age 30 for five years had an ICER of 72,000 EUR/LYG; the ICER for using oral contraceptives for five years as of age 50 was 450,000 EUR/LYG. Genetic testing for mutations in women at increased risk for carrying a mutation followed by risk-reducing surgery yielded ICERs below 40,000 EUR/QALY. Based on study results, preventive surgery in mutation carriers and genetic testing in women at increased risk for mutations are cost-effective. Except for high-risk women, screening using TVS and endometrial biopsy is not cost-effective and may lead to overtreatment. Model-based analyses indicate that future biomarker screening in women at increased risk for cancer may be cost-effective, dependent on high test accuracy and moderate test costs. Future research should reveal risk-adapted early detection and prevention strategies for endometrial cancer.

## 1. Introduction

Endometrial cancer is the sixth most common cancer in women and the most common female genital tract cancer in the developed world with rising incidences since 1990 [1,2]. North America and West Europe are the countries with highest incidences with age-standardized incidences of 20.5 and 22.2 per 100,000 women in the year 2018 [3,4]. Moreover, it is speculated that endometrial cancer incidences are rising due to the ageing population, and influencing life style factors (e.g., obesity, diabetes, lower parity) [5]. The large majority of endometrial cancer cases occur at ages over 50 years, with a peak incidence between 65 and 75 years of age [5,6]. The five-year survival rate depends on the cancer stage and is as high as 95% for local stage endometrial cancer, but as low as 18% for advanced stages [7]. 

Germline mutations such as Lynch syndrome may increase the lifetime risk for endometrial cancer. Women with Lynch syndrome have a 40–60% chance of developing endometrial cancer and a 3–14% chance of developing ovarian cancer [8,9]. Lynch syndrome accounts for less than one percent of all endometrial cancer cases [10]. Prevention strategies to reduce mortality in women with Lynch syndrome include chemoprevention and cancer risk-reducing surgery such as hysterectomy and bilateral salpingo-oophorectomy [11]. A large retrospective study demonstrated that none of the women with Lynch syndrome who underwent hysterectomy with or without bilateral salpingo-oophorectomy developed endometrial cancer (average follow-up time was 13.3 years after surgery) compared with 33% of the controls (average follow-up time 7.4 years) [12].

Unlike breast and cervical cancer, for which screening programs are available to the general population, endometrial cancer is most commonly diagnosed after endometrial biopsy in symptomatic patients (e.g., vaginal bleeding) [7,13]. In women with postmenopausal bleeding, the prevalence of endometrial cancer is about ten percent [14]. 

Identifying women at increased risk for endometrial cancer is crucially important for cancer prevention. Risk-tailored early detection and prevention strategies may have the potential to lower endometrial cancer incidence and mortality at reasonable costs. Benefits of screening and prevention need to be carefully balanced against both the clinical harms and the economic burden as health care resources are limited. Thus, it is critical to evaluate the evidence on cost-effectiveness of potential prevention or early detection strategies.

The purpose of this semi-quantitative systematic review was to identify and summarize current evidence on the long-term effectiveness and cost-effectiveness of different early detection and prevention strategies for endometrial cancer in various population subgroups.

## 2. Results

**Figure 1:**
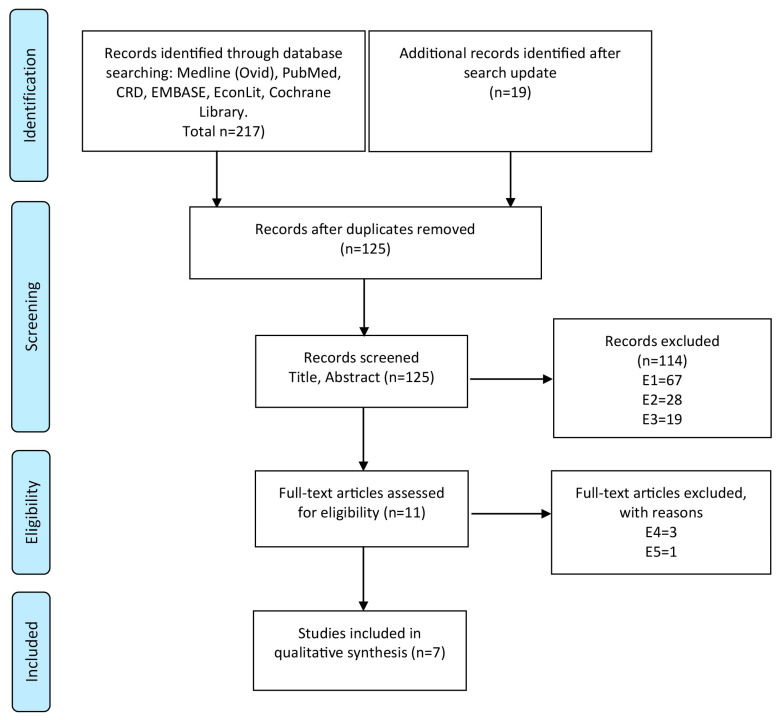
PRISMA flow diagram for the process of literature search and exclusion: Electronic data bases were searched for cost-effectiveness studies evaluating endometrial cancer screening and/or prevention strategies. Numbers of excluded studies are listed for each reason of exclusion. Exclusion criteria: E1—other diseases than endometrial cancer or already have endometrial cancer, recurrent cancer or metastases; E2—studies evaluating cost-effectiveness of other interventions (e.g., therapy of endometrial cancer); E3—not decision-analytic modelling studies; E4—not full health-economic studies (cost-effectiveness studies); E5—editorials, reviews, abstracts; E6—not in German or English language.

Out of 125 identified publication hits, seven studies [15,16,17,18,19,20,21] evaluating early detection and prevention strategies for endometrial cancer, met the inclusion criteria. Figure 1 shows the PRISMA flow diagram of the literature search including criteria for the selection of studies. 

The included studies were published between 2008 and 2017. All studies were performed for the health care system of the United States of America. No European study was found evaluating early detection or prevention strategies for endometrial cancer. The included studies showed heterogeneity regarding target population, study perspective, analytic approach, and compared intervention strategies.

We distinguished between (1) screening for endometrial cancer, (2) preventive interventions and (3) genetic screening for germline mutations. If studies reported more than one type of intervention, we separated the interventions and sorted them into these three categories. We found four studies reporting screening strategies for four different risk groups, five studies reporting risk-reducing interventions targeting women with high risk, and one study on genetic screening. Key characteristics of the included studies are summarized in Table 1. Application of the CHEERS checklist for reporting quality of cost-effectiveness studies resulted in similar scores for the included studies (18–21 out of 24 possible points) (Table 2). 

In the following paragraphs, the overall health-economic results are summarized for each category and target group presenting incremental cost-effectiveness ratios (ICERs) and incremental cost-utility ratios (ICURs) compared to the next non-dominated strategy.

### 2.1. Screening for Endometrial Cancer in Women with Different Risk Profiles

In total, four studies [17,18,19,20] evaluated different screening strategies for endometrial cancer in women with different cancer risk profiles (Table 3).

Of those, two studies [19,20] evaluated screening strategies including annual gynecological examination, transvaginal sonography (TVS), the biomarker CA-125, and endometrial biopsy in women with Lynch syndrome with 40–60% lifetime risk for endometrial cancer. Annual screening with TVS, the biomarker CA-125, and endometrial biopsy was considered cost-effective compared with no intervention for 30-year-old women with Lynch syndrome yielding an ICER of 43,600 EUR/LYG [19]. When considering quality of life, the ICUR of this screening strategy increased to 95,800 EUR/QALY compared with no intervention [19]. Annual screening with TVS, CA-125, and endometrial biopsy was less costly and more effective (i.e., dominant) compared with gynecological screening with TVS alone in the other study [20].

Two studies [17,18] evaluated screening strategies for endometrial cancer in obese (body mass index [BMI] > 30 kg/m²) women with at least 3% lifetime risk for endometrial cancer. One of these studies [17] evaluated the impact of specific test characteristics and costs of a potential new biomarker (hypothetical biomarker) on its potential clinical utility and cost-effectiveness in women at different risks for endometrial cancer. In particular, annual screening with a high-performing hypothetical serum test consisting of a multiple biomarker panel including prolactin (sensitivity: 0.98; specificity: 0.98) compared to annual endometrial biopsy or annual TVS was evaluated. Annual screening of obese women (age 45–80 years) with this hypothetical serum test yielded an ICER of 46,700 EUR/LYG compared with no screening [17]. Annual screening with TVS or endometrial biopsy alone was dominated, and therefore not cost-effective [17]. Starting screening at age 25 years generated a higher ICER [17]. In the other study, annual screening of obese women (age 30–80 years) with gynecological examination and endometrial biopsy alone was not cost-effective, having an ICER of over 1.5 million EUR/LYG compared with no screening [18]. 

Annual screening using the hypothetical serum biomarker panel in women older than 60 years with a breast cancer history at increased risk for endometrial cancer (6% lifetime risk), who had been using tamoxifen for up to five years, achieved an ICER of about 23,000 EUR/LYG compared with no screening [17]. Annual screening with TVS or with endometrial biopsy alone was less effective and more costly and therefore dominated. In women, from the general population (2.5% lifetime risk for endometrial cancer), annual screening between age 50 and 75 years using the hypothetical serum biomarker panel resulted in an ICER of about 68,400 EUR/LYG [17]. 

### 2.2. Risk-Reducing Interventions for Women at Increased or High Risk for Endometrial Cancer

Five studies [16,18,19,20,21] evaluated risk-reducing interventions in women at increased or high risk for endometrial cancer (Table 4).

Two of these studies [19,20] evaluated risk-reducing interventions including prophylactic surgery (at age 30 or 40 years) for the prevention of future endometrial and additionally ovarian or colon cancer in women with Lynch syndrome at 40–60% lifetime risk for endometrial cancer. Both studies also included screening strategies in their analyses, which were less effective and more costly, and therefore, dominated by prophylactic surgery (Table 4). In a 30-year-old women with Lynch syndrome, prophylactic hysterectomy and prophylactic bilateral salpingo-oophorectomy (PBSO) was considered cost-effective with an ICER of 2800 EUR/LYG compared with no intervention [19]. Prophylactic hysterectomy plus PBSO at age 40 with or without prior screening with endometrial biopsy was more costly and less effective, and was therefore dominated. When quality of life was taken into consideration, prophylactic hysterectomy and PBSO at age 40 dominated prophylactic hysterectomy and PBSO at age 30 with an ICUR of 5700 EUR/QALY compared with no intervention [19]. In the other study, prophylactic hysterectomy with PBSO was more effective and less costly compared with annual gynecologic screening including TVS with or without CA-125 [20].

One study [21] evaluated risk-reducing interventions in 40-year-old women with BRCA-1 mutations at high risk for uterine cancers additionally considering the risk for developing breast cancer and ovarian cancer. In these women, PBM plus PBSO with hysterectomy yielded more life years and was less costly compared with PBM plus PBSO alone, considering the risk for developing breast and ovarian cancer in addition to endometrial cancer (3.5% lifetime risk) [21]. When quality of life was taken into account, PBSO alone was more effective and was cost-effective with an ICUR of 13,000 EUR/QALY [21]. 

Two other studies [16,18] evaluated risk-reducing interventions to prevent endometrial cancer in obese women compared to no intervention or usual care.

Application of oral contraceptives for five years in 30-year-old obese women (lifetime risk for endometrial cancer of 3%) to prevent endometrial cancer was not cost-effective with an ICER of 458,800 EUR/LYG compared with no intervention [18]. However, using Levonorgestrel intrauterine devices for five years in 50-year-old obese women (age-dependent lifetime risk for endometrial cancer between 4% and 7%) yielded an ICER of 72,000 EUR/LYG in comparison with usual care [16].

### 2.3. Genetic Testing for Germline Mutations Followed by Risk-Reducing Interventions for Diagnosed Mutation Carriers 

Only one study [15] evaluated genetic testing for mutations in unaffected individuals having a family history of sporadic and/or Lynch syndrome-associated with endometrial and/or colorectal cancer (Table 5). Identified mutation carriers were then screened with TVS and endometrial biopsy (and colonoscopy to early detect colorectal cancer), and were offered prophylactic procedures such as total abdominal hysterectomy and PBSO (and polypectomy to prevent colorectal cancer). 

The ICURs varied widely depending on the age of the individual and the risk threshold for carrying a mutation, that was considered for genetic testing, with a range from 8200 EUR/QALY for performing genetic testing in populations with a risk of at least 10% to carry a mismatch repair gene mutation in individuals as of age 40, to over 7.4 million EUR/QALY for universal genetic testing in individuals as of age 20. The authors concluded that genetic testing for mismatch repair gene mutations in women age 25–35 years with a pretest risk of carrying a mutation higher than 5% may be cost-effective with an ICUR below 43,300 EUR/QALY gained compared with current practice.

**Figure 2:**
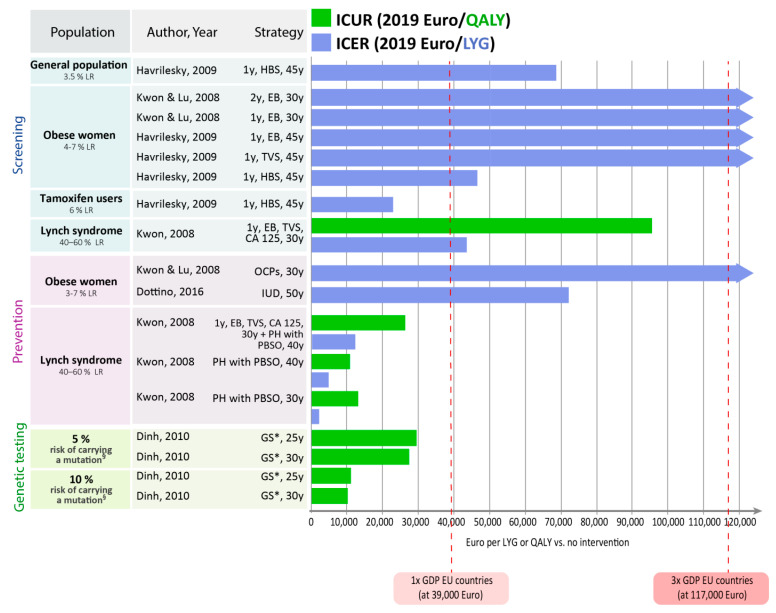
Cost-effectiveness results for each intervention in comparison to no intervention. EB: endometrial biopsy; GS: genetic screening + EB, TVS (plus colonoscopy to early detect colorectal cancer); HBS: hypothetical biomarker panel screening including prolactin (sensitivity and specificity of 0.98, both); IUD: Levonorgestrel intrauterine device; LR: lifetime risk; OCPs: oral contraceptive pills; PBSO: prophylactic bilateral salpingo-oophorectomy; PH: prophylactic hysterectomy; TVS: transvaginal sonography. § associated with Lynch syndrome. * Including risk-reducing surgery (e.g., hysterectomy, PBSO, polypectomy to prevent colorectal cancer) in mutation carrier.

While decision-making should be based on the incremental cost-effectiveness ratios reported above (Table 3, Table 4 and Table 5), determined in a step-wise approach [23], we additionally present cost-effectiveness and cost-utility ratios for each strategy in comparison to no intervention in Figure 2. This provides an overview over strategies across categories. As reference points, the figure shows EU GDP, but decisions should be made according to country-specific willingness-to-pay thresholds. 

The risk of developing endometrial cancer in the target population and the impact of interventions on the quality of life are the most important influence factors for cost-effectiveness results. The cost-effectiveness results of the different endometrial cancer early detection and prevention strategies depend mainly on the overall risk for developing endometrial cancer in the target populations, and on the intervention’s impact on the quality of life. 

In women at high risk for endometrial cancer (e.g., mutation carrier), risk-reducing prophylactic hysterectomy and PBSO compared with no intervention were highly cost-effective achieving ICERs and ICURs below the threshold of once the per capita GDP [19]. Although annual screening with TVS, CA-125, and endometrial biopsy compared with no intervention achieved also ICERs and ICURs below twice the per capita GDP (e.g., EUR 78,000), prophylactic surgery achieved much lower ICERs and ICURs [19]. Consideration of quality of life suggests that performing prophylactic surgeries at higher age results in a better quality of life in women [19]. 

Genetic testing for mutations in women at increased or high risk for carrying a mutation followed by risk-reducing surgery for mutation carriers compared with no intervention were highly cost-effective, achieving ICERs and ICURs even below the threshold of once the per capita GDP [15].

In women at increased risk for endometrial cancer (e.g., obese women), intrauterine devices with Levonorgestrel for five years at age 50 achieved an ICER below twice the per capita GDP, which can be considered cost-effective [16]. Whereas, oral contraceptive pills for five years in women at age 30 years were not cost-effective [18]. Further, annual screening with TVS or endometrial biopsy in women at increased risk for endometrial cancer (e.g., obese women or tamoxifen users) was not cost-effective, achieving ICERs above three times the per capita GDP [17,18]. 

An analysis assuming a hypothetical future screening test with high test accuracy (sensitivity: 98%, specificity: 98%) reported this screening test to be highly cost-effective (once the per capita GDP) in women at increased risk for endometrial cancer (e.g., women 61–80 years with a breast cancer history, who have been using tamoxifen for up to five years) and to be cost-effective (twice the per capita GDP) in postmenopausal women from the general population or in obese women as of age 45 years [17].

## 3. Discussion

We conducted a systematic literature search with the objective to give an overview of the current evidence on the effectiveness and cost-effectiveness of screening and prevention strategies for endometrial cancer. According to our study, the cost-effectiveness is influenced strongly by the risk level in the chosen target population. It also depends to a large extent on the intervention’s impact on quality of life and finally on the willingness-to-pay threshold of the specific country or health care system.

Study results suggest that preventive surgery, for example, hysterectomy plus PBSO, may be considered highly cost-effective in women at high risk (e.g., mutation carriers) for developing endometrial cancer. In women at increased risk, younger age at intervention (e.g., 30 versus 40 years) results in very low ICERs, but in higher ICURs when including quality of life. This points to the importance of including quality of life in the evaluation in addition to mortality reductions and remaining life expectancy. As prophylactic surgery may have severe side effects, which strongly impact the quality of life of the individual woman (e.g., preterm menopause and infertility), the age at which the prophylactic surgery is offered should be chosen wisely. For women at high risk for endometrial cancer not willing to undergo prophylactic surgery, annual screening with TVS, CA-125, and endometrial biopsy may also be a cost-effective alternative. One study on genetic testing for mutations in women with familial risk found a 5% threshold for pre-test risk of carrying a mutation above which genetic testing is highly cost-effective if followed by risk-reducing surgery for mutation carriers. 

In obese women at increased risk for endometrial cancer, Levonorgestrel to prevent endometrial cancer may be considered cost-effective when treating women as of age 50 for five years. In contrast, treating obese women as of age 30 with oral contraceptives for five years cannot be considered cost-effective, neither can be screening approaches with annual TVS or endometrial biopsy.

In women at average risk for endometrial cancer, no reliable early detection, and no prevention strategy are currently available. In our review, one study reported that annual screening with a high-performing hypothetical test (98% sensitivity and 98% specificity) may be effective and cost-effective to early detect endometrial cancer in women from the general population as well as in women at increased cancer risk. These results emphasize that a future screening test for endometrial cancer not only needs to detect malignancies at a very high rate, but also needs to be very accurate in order to avoid unnecessary treatment associated with false-positive results.

Current evidence advises against endometrial cancer screening in asymptomatic women in the general population. Presently applied screening tests such as TVS or endometrial biopsy have not yet demonstrated to reduce endometrial cancer mortality. Controversies still exist on what endometrial thickness threshold to apply when deciding on biopsy. Using the same threshold for asymptomatic women as is used for women with abnormal vaginal bleeding (4 mm cut-off) [24] will lead to poor sensitivity and a high number of false-positive rates. In our review, one decision-analytic study [17] applied a cut-off value of 5 mm in annual TVS screening, but results were not cost-effective for the asymptomatic general population. New cut-off values with higher sensitivity of TVS have been evaluated by Jacobs et al. [25] and Smith-Bindman [26]. However, there is no evidence suggesting a reduction in cancer-related mortality, yet. As such, TVS cannot be implemented for endometrial cancer screening for now, and further research as well as a consensus for the recommended cut-off are needed. 

Today’s guidelines recommend either prophylactic surgery or annual screening with TVS and endometrial biopsy as of age 35 years for women with Lynch syndrome. In modeling studies, both screening and preventive surgery in women as of age 30 years were shown to be cost-effective, but only when quality of life was not considered. Consideration of quality of life is of high importance though as each early detection or prevention strategy might have possible implications for the physical and psychological health of women. Invasive interventions such as an endometrial biopsy or a prophylactic surgery (e.g., hysterectomy and PBSO) often have moderate to severe side effects. Preventive surgeries may lead to infertility and artificial menopause in women at childbearing age. Preventive surgeries may affect body image negatively, cause disturbances in sexual relationships, and generate psychological distress [27]. For women with increased or high risk, the decision about preventive interventions is difficult and highly personal. It is important that clinicians are qualified to inform and consult carefully and communicate clearly and sensitively with women in the decision process. 

To our best knowledge, this is the first study systematically summarizing current evidence on the cost-effectiveness of early detection or preventive strategies for endometrial cancer for women with different risk profiles. 

Our study has particular strengths. One specific strength of our review is that we performed additional calculations with the published data such as the incremental cost-effectiveness ratios based on the reported effects and cost of the individual studies. All ICERs/ICURs are consistently calculated in the step-wise manner considering dominance and extended dominance as is necessary to be relevant for decision-making [23,28]. To facilitate quantitative comparison across studies, all cost data have been converted to 2019 euros, which is a particular strength of this semi-quantitative review. In addition, we provide a visual overview of results for all strategies when compared to a common baseline. While reporting results, we highlighted the role of risk-adaptation for screening and prevention strategies for endometrial cancer, and we performed a critical assessment of the model aspects.

Decision-analytic models, as a simplification of reality, have their limitations. Analyses outcomes will depend greatly on the model assumptions, methodology, and structure of the model [29]. 

First, the studies varied significantly in the study perspective, time horizon, and in the evaluated interventions, which makes comparison between studies difficult. Compared interventions in different studies for the same population subgroup differ greatly in the type and frequency of the interventions. Three studies reported to adopt a societal perspective but included only direct medical costs. Incomplete cost assessments (e.g., excluding indirect costs) may lead either to underestimated or overestimated ICERs and ICURs. Thus, these study findings may be biased or rather considered as findings adopting a payer’s perspective. 

Second, an important limitation of most of the studies was a lack of reporting model validations. International guidelines for modeling recommend that the “validation of a model should include an evaluation of face validity of the structure, evidence, problem formulation, and results of the model.” Models that are not validated may bear a potential risk of bias [30].

One study [20] used a decision tree model to evaluate the cost-effectiveness of preventive surgery compared to annual gynecological examination in high-risk women. Although some questions may be answered with this approach, results of this analysis should be interpreted cautiously. A decision tree approach does not easily allow considering time to an event and a change in probabilities over time. Decision trees are mainly useful for modeling diseases and intervention effects with short time horizons [31]. Some effects of screening interventions, especially lead-time and overdiagnosis, as well as overtreatment, cannot fully be represented in decision tree models.

Third, not all studies considered quality of life, and none of the included studies considered the anxiety that women might experience when they receive a positive test result. While in some studies the impact of a preventive intervention such as surgery on quality of life was considered, the impact of receiving a positive test result itself was not incorporated in any of the analyses. Studies not including quality of life measures in women receiving a positive test result and/or undergoing surgery might not be able to thoroughly evaluate the trade-off between clinical benefits and psychological harms of screening.

Fourth, in modeling studies evaluating screening strategies, false-positive test results leading to unnecessary surgeries (e.g., hysterectomy, PBSO) could potentially have a positive effect if they prevent endometrial cancer in women who would have developed it later in life (and in the model) without the surgery. Studies neglecting the effect on quality of life caused by positive test results and necessary or unnecessary surgery may overestimate benefits and underestimate cost-effectiveness ratios. Both may lead to falsely favoring strategies with lower specificity and therefore more false-positive results and unnecessary surgeries. A similar effect may occur in modeling studies when not all downstream costs of positive test results are carefully included.

Finally, it is crucial that all relevant strategies (variations on screening intervals, start and stop age) are included as comparators in the analysis and that cost-effectiveness ratios are reported for each non-dominated strategy and always in comparison to the next best non-dominated strategy [32]. This principle was not always followed in the evaluated studies.

Besides the limitations of individual studies, our review has limitations of its own. Our search for studies was focused on cost-effectiveness studies based on a decision-analytic model, evaluating screening and prevention for endometrial cancer in asymptomatic women. We may have missed evidence from effectiveness studies not reporting costs. In addition, our results may be not representative for women presenting with symptoms such as abnormal bleeding (e.g., vaginal bleeding in postmenopausal women). While we searched in a range of relevant electronic databases, we may have missed further studies in the gray literature. Publications in languages other than English or German were also not considered. 

Future research should consider all of the issues discussed above and may also focus on risk-adapted strategies to early detect and prevent endometrial cancer considering genomics, epigenetics, and lifestyle information, including individual characteristics (e.g., age, time since menopause, reproductive factors). To date, only a few endometrial cancer prediction models have been developed especially for asymptomatic women [33]. Likewise, there are currently no established tests available to predict the individual risk to develop endometrial cancer—especially poor prognostic cancer—which may help to tailor different screening and prevention strategies according to women’s risk. This kind of biomarker test would be of critical value for the implementation of risk-stratified interventions. Further, the evaluation of public health programs to facilitate lifestyle changes for specific women at increased risk would be of interest. In general, future studies should include all relevant invasive (e.g., prophylactic surgery) and non-invasive comparator strategies for the prevention or early-detection of endometrial cancer in order to make a comprehensive comparison. 

This review can guide further research into risk-adapted, personalized early detection and prevention strategies for endometrial cancer and provide guidance for informed decisions in health care resource allocation. 

## 4. Materials and Methods

This semi-quantitative systematic review consists of several steps. In the first step, we systematically searched for studies evaluating both the clinical long-term effectiveness and cost-effectiveness of early detection and prevention strategies for endometrial cancer in the electronic databases Medline (Ovid and PubMed), Embase (Ovid), the Cochrane Library, CRD databases (NHS EED, DARE, HTA Database), and EconLit (last update: January 2020). The search codes were developed separately for each database using medical subject headings (MeSH) and search term combinations for endometrial cancer, detection or prevention, effectiveness, costs, and modeling (Table S1). Additionally, we screened the reference lists of identified reviews for relevant literature. All references were imported into a literature database (Endnote version X7, Thomson Corp., Stamford, CT, USA). Two authors (AG, GS) screened reference titles and abstracts for relevant articles. Subsequently, references were selected based on a-priori defined inclusion and exclusion, after reading the full-text documents. If there was any discrepancy among reviewers, a third reviewer (ACF) took the decision. The Consolidated Health Economic Evaluation Reporting Standards (CHEERS) checklist [34] was applied to all included studies (Table 2).

We included decision-analytic modeling studies assessing both the long-term effectiveness and the cost-effectiveness of different early detection and prevention strategies for endometrial cancer, reporting outcome measures such as quality-adjusted life-years gained (QALY), life-years gained (LYG), incremental cost-utility ratios (ICUR; in cost/QALY), or incremental cost-effectiveness ratios (ICER; in cost/LYG). We included studies with a time horizon sufficiently long to reflect that cancer and preventive interventions affect life expectancy and overall costs [35,36]. We excluded studies in languages other than English or German, unsystematic reviews, editorials, letters, abstracts, and studies which were not full health-economic evaluations or evaluating follow-up or treatment strategies, as well as costing studies not using a decision-analytic model.

In the second step, we systematically extracted and summarized the following information in standardized evidence tables: model analytic framework and characteristics (target population, study type, perspective, time horizon, discount rate, model type and simulation type, sensitivity analysis, model validation), clinical effectiveness, and cost-effectiveness results of compared strategies. In cases where ICER or ICUR were not reported in the included study, we calculated ICER and ICUR based on the reported study data if possible. 

The third step served the comparison across countries, currencies, and study years. We converted all costs into 2019 euros using the gross domestic product (GDP) purchasing power parities (PPP) to adjust for the country-specific purchasing power and the consumer price index (CPI) of Europe to adjust for inflation [37,38,39]. 

In the fourth step, in order to quantitatively compare cost-effectiveness measures across studies, we calculated ICER or ICUR compared to the next non-dominated strategy, if necessary and if data were available. Strategies are considered dominated if they provide less health benefit at higher costs when compared with any other strategy [40,41]. Similarly, dominated strategies should not be considered by decision-makers and no ICER is calculated. Furthermore, extended dominance is applied to eliminate strategies, for which costs and benefits are dominated by a mix of two other alternatives. 

In the fifth step, we performed the judgment of cost-effectiveness. We did not use a global willingness-to-pay (WTP) threshold for incremental cost-effectiveness ratios, as these thresholds vary across different countries [42]. In this review, the reported incremental cost-effectiveness of different studies is based on the reported truly (i.e., stepwise) incremental cost-effectiveness and cost-utility ratios, that is, always comparing the evaluated strategy to the next effective non-dominated strategy.

Finally, we calculated the ICER or ICUR compared to no intervention or standard of care as a simple descriptive point of reference for all included studies, if data were available. This step served to visualize the cost-effectiveness measures across different risk groups, interventions, studies, and countries. Only for this visualization, we used the World Health Organization (WHO) recommended willingness-to-pay threshold of one to three times the GDP per capita for a specific country [43,44] to show the potential lower and upper willingness-to-pay limits for countries of the European Union in comparison with the ICER or ICUR. Thus, when visualizing our results compared to no intervention or standard care, we show the lower and upper limit of this range using the 2019 average GDP per capita in 28 countries of the European Union [37], which is approximately EUR 39,000 to EUR 117,000. This visualization serves as a mere descriptive point of orientation for European countries, as decision-making should be based on country-specific willingness-to-pay thresholds and on truly stepwise incremental cost-effectiveness ratios [23]. 

All abbreviations used are summarized in Table S2.

## 5. Conclusions

Based on study results, genetic testing in women at increased risk for mutations followed by preventive surgery in mutation carriers as well as preventive surgery in known mutation carriers can be considered effective and cost-effective. Except for women with diagnosed Lynch syndrome, screening strategies using transvaginal sonography or endometrial biopsy are likely not cost-effective. Screening with transvaginal sonography or endometrial biopsy in women at average risk may lead to overtreatment and cannot be considered cost-effective. Promising model-based results suggest that future biomarker screening in asymptomatic women at increased risk for cancer may be cost-effective if the test accuracy is sufficiently high and test costs are moderate. Future research is needed on risk-adapted early detection and prevention strategies for endometrial cancer, integrating harms of intervention and considering overdiagnosis. Future trials may investigate new biomarkers for preventive strategies based on genomics, epigenetics, and lifestyle information.

## Figures and Tables

**Table 1:** Characteristics of Cost-Effectiveness Studies Evaluating Endometrial Cancer Screening and/or Prevention Strategies Sorted by Intervention Category.

Study, Publication Year, Country	Objectives, Compared Strategies, Target Population	Model Type, Analysis Approach, Time Horizon	Study Type, Perspective, Costs, Discount Rate, Index Year, Currency	Outcome Measures	Sensitivity Analyses	Model Validation
Havrilesky, 2009, USA [17]	Evaluation of screening strategies. Annual EB, annual TVS, and annual serum screening with a hypothetical biomarker vs. no screening, (1) increased risk: obese women and (2) average risk: women from the general population	Markov model, cohort simulation, lifetime	CEA, societal, direct medical costs (indirect costs were not explicitly mentioned), 3% (costs and effects) 2006, U.S. dollars	LY, costs, ICER (costs/LYG), ACER (costs/LY)	Scenario analyses (Univariate)	n.r.
Kwon and Lu, 2008, USA [18]	Evaluation of screening and preventive interventions, no prevention (reference strategy), oral contraceptive pills for 5 yrs, annual screening with EB from age 30, biennial screening from age 30, increased risk: obese women, age 30 yrs	Markov model, cohort simulation, lifetime	CEA, societal direct and indirect health care costs, 3% (costs and effects) 2006, U.S. dollars	LY, costs, ICER (costs/LYG)	Scenario analyses (Univariate)	n.r.
Kwon et al., 2008, USA [19]	Evaluation of prophylactic surgery and screening, prophylactic surgery, annual screening with EB, TVS, and CA-125 vs. no prevention, high-risk: women with Lynch syndrome age 30+ yrs	Markov model, cohort simulation, lifetime (40 yrs)	CUA, societal, direct and indirect costs, 3% (costs and effects) 2006, U.S. dollars	LY, QALY, costs, ICUR (costs/QALY gained)	Deterministic, probabilistic	n.r.
Yang, 2011, USA [20]	Evaluation of prophylactic surgery and screening, risk-reducing hysterectomy with PBSO vs. annual gynecologic examination + TVS (and CA- 125, EB), high-risk: women with Lynch syndrome age 30+ yrs	Decision tree, cohort simulation, lifetime	CUA, societal, direct costs (only health care costs reported), 3% (costs and effects) 2010, U.S. dollars	QALY, lifetime costs, ICUR (costs/QALY gained)	Deterministic univariate and probabilistic multivariate	n.r.
Havrilesky, 2017, USA [21]	Evaluation of prophylactic surgery. PBM+PBSO alone vs. PBM+PBSO with hysterectomy. High-risk: BRCA-1 mutation carriers with no cancer, age 40 yrs, following PBM+PBSO	Markov model, cohort simulation, lifetime	CEA, health care sector, direct costs, 3% (costs and effects) 2015, U.S. dollars	LY, ICER (costs/LYG), QALY (scenario)	One-way and Monte Carlo probabilistic, scenario analyses	n.r.
Dottino, 2016, USA [16]	Evaluation of preventive intervention, Levonorgestrel intrauterine device vs. usual care. Increased risk: obese women, age 50 yrs	Markov model, cohort simulation, lifetime	CEA, payer, direct costs, 3% (costs and effects) 2015, U.S. dollars	ICER (costs/LYG)	One-way, two-way and Monte Carlo probabilistic	n.r.
Dinh, 2010, USA [15]	Evaluation of annual genetic screening strategies for Lynch syndrome, (1) screening of unaffected individuals via demographic and family histories, and offering genetic testing to those individuals whose risks for carrying a mutation exceed a selected threshold vs. standard of care. (2) Universal genetic testing, both strategies were compared to current practice of genetic testing for persons with family history of Lynch syndrome and to each other. General population	Mathematical equation model (Archimedes model), microsimulation, lifetime	CUA, societal, direct medical costs (indirect costs were not explicitly mentioned), 3% (costs and effects) 2009, U.S. dollars	LY, QALYs, costs, ICUR (costs/QALY)	Scenario analyses (Univariate SA),	Against observed data

ACER: average cost-effectiveness ratio (the average cost-effectiveness ratio is the ratio of the cost to benefit of an intervention without reference to a comparator), EB: endometrial biopsy, ICER: incremental cost-effectiveness ratio, ICUR: incremental cost-utility ratio, LY: life year, n.r.: not reported, PBM: prophylactic mastectomy, PBSO: prophylactic bilateral salpingo-oophorectomy, QALY: quality-adjusted life year, SA: sensitivity analyses, TVS: transvaginal sonography; yrs: years.

**Table 2:** CHEERS checklist for included studies.

Author	Year	Title	Abstract	Introduction	Target Population and Subgroups	Setting and Location	Study Perspective	Comparators	Time Horizon	Discount Rate	Choice of Health Outcome	Measurement of Effectiveness	Measurement and Valuation of Preference-Based Outcomes	Estimating Resources and Costs	Currency, Price Date, and Conversion	Choice of Model	Assumptions	Analytic Methods	Study Parameters	Incremental Costs and Outcomes	Characterizing Uncertainty	Characterizing Heterogeneity	Discussion	Source of Funding	Conflicts of Interest	Points (out of 24)	Items Present in %
Havrilesky et al. [21]	2017	Y	Y	Y	Y	Y	Y	Y	Y	Y	Y	Y	N	Y	Y	Y	Y	N	Y	Y	Y	N	Y	N	Y	**20**	**83**
Dottino et al. [16]	2016	Y	Y	Y	Y	Y	Y	Y	Y	Y	Y	Y	N	Y	Y	Y	Y	N	Y	Y	Y	N	Y	N	Y	**20**	**83**
Yang et al. [20]	2011	Y	Y	Y	Y	N	N	Y	Y	Y	Y	Y	N	Y	Y	Y	Y	N	Y	Y	Y	Y	Y	Y	Y	**20**	**83**
Dinh et al. [15]	2011	Y	Y	Y	Y	Y	N	Y	Y	Y	Y	Y	N	Y	Y	Y	Y	N	Y	Y	Y	Y	Y	Y	Y	**21**	**88**
Havrilesky et al. [17]	2009	Y	Y	Y	Y	Y	N	Y	Y	Y	Y	Y	N	Y	Y	Y	Y	N	Y	Y	Y	Y	Y	N	N	**19**	**79**
Kwon et al. [19]	2008	Y	Y	Y	Y	Y	Y	Y	Y	Y	Y	Y	N	Y	Y	Y	Y	N	Y	Y	Y	Y	Y	N	N	**19**	**79**
Kwon and Lu [18]	2008	Y	Y	N	Y	Y	Y	Y	Y	Y	Y	Y	N	Y	Y	Y	Y	N	Y	Y	Y	N	Y	N	N	**18**	**75**
Number of studies missing item (out of 7 studies)		0	0	1	0	1	3	0	0	0	0	0	7	0	0	0	0	7	0	0	0	3	0	5	3		
Studies missing item (%)		0	0	14	0	14	43	0	0	0	0	0	100	0	0	0	0	100	0	0	0	43	0	71	43		

Y: Yes, present; N: No, not pres.

**Table 3:** Discounted Costs, Life Years, Quality-Adjusted Life-Years (QALYs), and Incremental Cost-Effectiveness Ratios of Endometrial Cancer Screening Strategies in Women with Different Risk Profiles.

Study, Country, Currency	Target Population Compared Strategies	Over no Intervention or Current Practice					Compared to Next Non-Dominated				
		Incr. Costs in 2019 Euro (in 2019 USD)	Incr. LY	Incr. QALY	ICER in 2019 Euro/LYG (in 2019 USD/LYG)	ICUR in 2019 Euro/QALY (in 2019 USD/ QALY)	Incr. Costs in 2019 Euro ^a^ (in 2019 USD)	Incr. LYG ^a^	Incr. QALY^a^	ICER in 2019 EUR/ LYG ^a^ (in 2019 USD/LYG)	ICUR in 2019 EUR/ QALY ^a^ (in 2019 USD/QALY)
Kwon et al., 2008, USA, USD [19]	Target population: women with Lynch syndrome, age 30 yrs and older, 40–60% lifetime risk for endometrial cancer (colon and endometrial cancer)										
	Strategies:										
	No intervention	-	-	-	-	-	-	-	-	-	-
	EB, TVS, CA-125, age 30+, 1 yr	19,592 (21,930)	0.45	0.20	43,586 (48,787)	95,804 (107,235)	19,592 (21,930)	0.45	0.20	43,586 (48,787)	95,804 (107,235)
Yang et al., 2011, USA, USD [20]	Target population: high-risk: Lynch syndrome, women age 30 yrs, 40–60% lifetime risk of endometrial cancer (colon and endometrial cancer)										
	Strategies:										
	Exam, TVS, CA- 125, EB, age 30+, 1 yr	-	n.r.	-	n.r.	-	-	n.r.	-	n.r.	-
	Exam, TVS, age 30+, 1 yr	Dom	n.r.	Dom	n.r.	Dom	Dom	n.r.	Dom	n.r.	Dom
Havrilesky, 2009, USA, USD [17]	Target population: women with a history of breast cancer using tamoxifen for 5 yrs, age 61 to 80 yrs, 6% lifetime risk for endometrial cancer										
	Strategies:										
	No intervention	-	-	-	-	-	-	-	-	-	-
	HBS, age 61–80, 1 yr	n.r.	n.r.	n.r.	22,988 (25,730)	n.r.	n.r.	n.r.	n.r.	22,988 (25,730)	n.r.
	TVS, age 61–80, 1 yr	n.r.	n.r.	n.r.	n.r.	n.r.	Dom	Dom	n.r.	Dom	n.r.
	EB, age 61–80, 1 yr	n.r.	n.r.	n.r.	n.r.	n.r.	Dom	Dom	n.r.	Dom	n.r.
Havrilesky, 2009, USA, USD [17]	Target population: obese (>BMI 30 kg/m²) women, age 45 to 80 yrs, from 4% to 7% (age dependent) lifetime risk for endometrial cancer										
	Strategies:										
	No intervention	-	-	-	-	-	-	-	-	-	-
	HBS, age 45–80, 1 yr	541 (606)	0.0116	n.r.	46,678 (52,247)	n.r.	541 (606)	0.0116	n.r.	46,678 (52,247)	n.r.
	TVS, age 45–80, 1 yr	2892 (3237)	0.0105	n.r.	275,417 (308,280)	n.r.	Dom	Dom	n.r.	Dom	n.r.
	EB, age 45–80, 1 yr	3453 (3865)	0.0092	n.r.	375,283 (420,062)	n.r.	Dom	Dom	n.r.	Dom	n.r.
Kwon and Lu, 2008, USA, USD [18]	Target population: obese (> BMI 30 kg/m²) women, age 30 to 80 yrs, 3% lifetime risk for endometrial cancer										
	Strategies:										
	No intervention	-	-	-	-	-	-	-	-	-	-
	Exam +EB, age 30–80, 2 yrs	18,409 (20,606)	0.0100	n.r.	1,840,907 (2,060,562)	n.r.	18,409 (20,606)	0.0100	n.r.	1,840,907 (2,060,562)	n.r.
	Exam +EB, age 30–80, 1 yr	29,551 (33,077)	0.0172	n.r.	1,718,084 (1,923,084)	n.r.	11,142 (12,471)	0.0072	n.r.	1,547,496 (1,732,142)	n.r.
Havrilesky, 2009, USA, USD [17]	Target population: general population, women age 50 to 75 yrs, 2.5% lifetime risk for endometrial cancer										
	Strategies:										
	No intervention	-	-	-	-	-	-	-	-	-	-
	HBS, age 50–75, 1 yr	n.r.	n.r.	n.r.	68,392 (76,552)	n.r.	n.r.	n.r.	n.r.	68,392 (76,552)	n.r.
	TVS age 50–75, 1 yr	n.r.	n.r.	n.r.	n.r.	n.r.	n.r.	n.r.	n.r.	Dom	n.r.
	EB, age 50–75, 1 yr	n.r.	n.r.	n.r.	n.r.	n.r.	n.r.	n.r.	n.r.	Dom	n.r.

Dom: dominated, EB: endometrial biopsy, Ext dom: extended dominance, HBS: hypothetical biomarker panel screening including prolactin (sensitivity and specificity of 0.98, both), ICER: incremental cost-effectiveness ratio, ICUR: incremental cost-utility ratio, LYG: life years gained, QALY: quality-adjusted life year, TVS: transvaginal sonography, yrs: years. ^a^ calculated versus next (non-dominated) strategy.

**Table 4:** Discounted Costs, Life Years, QALYs, and Incremental Cost-Effectiveness Ratios of Risk-Reducing Interventions for Women at Increased or High Risk for Endometrial Cancer.

Study, Country, Currency	Target Population Compared Strategies	Over no Intervention or Current Practice					Compared to Next Non-Dominated				
		Incr. Costs in 2019 Euro (in 2019 USD)	Incr. LY	Incr. QALY	ICER in 2019 EUR/LYG (in 2019 USD/LYG)	ICUR in 2019 EUR/QALY (in 2019 USD/ QALY)	Incr. Costs in 2019 Euro ^a^ (in 2019 USD)	Incr. LYG ^a^	Incr. QALY ^a^	ICER in 2019 EUR/ LYG ^a^ (in 2019 USD/LYG)	ICUR in 2019 EUR/ QALY ^a^ (in 2019 USD/QALY)
Kwon et al., 2008, USA, USD [19]	Target population: women with Lynch syndrome, age 30 yrs and older, 40–60% lifetime risk for endometrial cancer, 42% for colon cancer (colon and endometrial cancer)										
	Strategies:										
	No intervention	-	-	-	-	-	-	-	-	-	-
	PBSO + hysterectomy at 30 yrs	5555 (6218)	1.996	0.353	2783 (3115)	15,724 (17,600)	5555 (6218)	1.996	0.353	2783 (3115)	Ext dom
	PBSO + hysterectomy at 40 yrs	6304 (7056)	1.053	0.485	5984 (6699)	13,003 (14,555)	749 (838)	Dom	0.132	Dom	5695 (6375)
	EB, TVS, CA-125, age 30–39, 1 yr; PBSO + hysterectomy, age 40	13,716 (15,353)	1.118	0.518	12,272 (13,736)	26,459 (29,616)	7412 (8297)	Dom	0.034	Dom	220,599 (246,920)
	EB, TVS, CA-125, age 30+, 1 yr	19,592 (21,930)	0.45	0.20	43,586 (48,787)	95,804 (107,235)	Dom	Dom	Dom	Dom	Dom
Yang et al., 2011, USA, USD [20]	Target population: high-risk: Lynch syndrome, women age 30 yrs, 40–60% lifetime risk of endometrial cancer (colon and endometrial cancer)										
	Strategies:										
	PBSO+hysterectomy, at 30 yrs	n.r.	n.r.	n.r.	n.r.	n.r.	n.r.	n.r.	n.r.	-	-
	Exam, TVS, CA- 125, EB, age 30+, 1 yr	n.r.	n.r.	n.r.	n.r.	n.r.	Dom	n.r.	Dom	n.r.	Dom
	Exam, TVS, age 30+, 1 yr	n.r.	n.r.	n.r.	n.r.	n.r.	Dom	n.r.	Dom	n.r.	Dom
Havrilesky et al., 2017, USA, USD [21]	Target population: BRCA-1 mutation carriers, women age 40 yrs with no cancer (following PBM+PBSO), 3.5% lifetime risk for endometrial cancer										
	Strategies:										
	PBM + PBSO + hysterectomy, at 40 yrs	n.r.	n.r.	n.r.	n.r.	n.r.	n.r.	n.r.	n.r.	-	-
	PBM+PBSO, at 40 yrs	n.r.	n.r.	n.r.	n.r.	n.r.	Dom	Dom	n.r.	Dom	12,989 (14,539)
Dottino, 2016, USA USD [16]	Target population: obese women, age 50 yrs, from 4% to 7% (age dependent) lifetime risk for endometrial cancer										
	Strategies:										
	No intervention	-	-	-	-	-	-	-	-	-	-
	Levonorgestrel, age 50–55	n.r.	n.r.	n.r.	71,992 (80,582)	n.r.	n.r.	n.r.	n.r.	71,992 (80,582)	n.r.
Kwon and Lu, 2008, USA, USD [18]	Target population: obese (> BMI 30 kg/m²) women, age 30–80 yrs, 3% lifetime risk for endometrial cancer										
	Strategies:										
	No intervention	-	-	-	-	-	-	-	-	-	-
	OCP, age 30–35	2982 (3338)	0.0065	n.r.	458,780 (513,521)	n.r.	2982 (3338)	0.0065	n.r.	458,780 (513,521)	n.r.
	Exam, EB, age 30–80, 2 yrs	18,409 (20,606)	0.0100	n.r.	1,840,907 (2,060,562)	n.r.	Ext dom	Ext dom	n.r.	Ext dom	n.r.
	Exam, EB, age 30–80,1 yr	29,551 (33,077)	0.0172	n.r.	1,718,084 (1,923,084)	n.r.	26,569 (29,739)	0.0107	n.r.	2,483,081 (2,779,361)	n.r.

Dom: dominated, EB: endometrial biopsy, Ext dom: extended dominance, ICER: incremental cost-effectiveness ratio, ICUR: incremental cost-utility ratio, LYG: life years gained, OCP: oral contraceptive pills, PBM: prophylactic mastectomy, PBSO: prophylactic bilateral salpingo-oophorectomy, QALY: quality-adjusted life year, TVS: transvaginal sonography, yrs: years. ^a^ calculated versus next (non-dominated) strategy.

**Table 5:** Discounted Costs, Life Years, QALYs, and Incremental Cost-Effectiveness Ratios of Genetic Testing for Germline Mutations Followed by Risk-Reducing Interventions for Diagnosed Mutation Carriers.

Study, Country, Currency	Target Population Compared Strategies	Over no Intervention or Current Practice					Compared to Next Non-Dominated				
		Incr. Costs in 2019 Euro (in 2019 USD)	Incr. LY	Incr. QALY	ICER in 2019 EUR/LYG (in 2019 USD/LYG)	ICUR in 2019 EUR/QALY (in 2019 USD/ QALY)	Incr. Costs in 2019 Euro ^a^ (in 2019 USD)	Incr. LYG ^a^	Incr. QALY ^a^	ICER in 2019 EUR/LYG ^a^ (in 2019 USD/LYG)	ICUR in 2019 EUR/QALY ^a^ (in 2019 USD/QALY)
Dinh, 2010, USA, USD [15]	Target population: general population, women age 20–40 yrs, different lifetime risks for endometrial cancer. Costs and QALYs are per 100,000 persons.										
	Strategies: standard of care vs. genetic testing at different ages and risk thresholds (%) of carrying mutation ^b,c^										
	*Genetic testing above 10% risk of having the mutation at different ages*										
	40 yrs	319,392 (357,501)	n.r.	45	n.r.	7098 (7944)	319,392 (357,501)	n.r.	45	n.r.	8246 (9229)
	35 yrs	532,319 (595,835)	n.r.	56	n.r.	(10,640)	212,928 (238,334)	n.r.	11	n.r.	10,999 (12,311)
	30 yrs	638,783 (715,002)	n.r.	63	n.r.	10,139 (11,349)	106,464 (119,167)	n.r.	7	n.r.	15,887 (17,782)
	25 yrs	745,247 (834,169)	n.r.	67	n.r.	11,123 (12,450)	106,464 (119,167)	n.r.	4	n.r.	39,677 (44,411)
	20 yrs	958,175 (1,072,504)	n.r.	69	n.r.	13,887 (15,544)	Ext dom	n.r.	Ext dom	n.r.	Ext dom
	*Genetic testing above 5% risk of having the mutation at different ages*										
	40 yrs	2,874,524 (3,217,511)	n.r.	102	n.r.	28,182 (31,544)	Ext dom	n.r.	Ext dom	n.r.	Ext dom
	35 yrs	3,300,379 (3,694,179)	n.r.	125	n.r.	26,403 (29,553)	2,555,132 (2,860,009)	n.r.	58	n.r.	43,272 (48,435)
	30 yrs	3,726,235 (4,170,847)	n.r.	135	n.r.	27,602 (30,895)	Ext dom	n.r.	Ext dom	n.r.	Ext dom
	25 yrs	4,365,018 (4,885,849)	n.r.	147	n.r.	29,694 (33,237)	1,064,639 (1,191,671)	n.r.	22	n.r.	47,416 (53,073)
	20 yrs	5,003,801 (5,600,852)	n.r.	151	n.r.	33,138 (37,092)	Ext dom	n.r.	Ext dom	n.r.	Ext dom
	*Genetic testing above 2.5% risk of having the mutation at different ages*										
	40 yrs	12,562,734 (14,061,713)	n.r.	220	n.r.	57,103 (63,917)	Ext dom	n.r.	Ext dom	n.r.	Ext dom
	35 yrs	13,733,837 (15,372,551)	n.r.	266	n.r.	51,631 (57,792)	9,368,819 (10,486,701)	n.r.	119	n.r.	78,808 (88,211)
	30 yrs	15,863,114 (17,755,892)	n.r.	288	n.r.	55,080 (61,652)	Ext dom	n.r.	Ext dom	n.r.	Ext dom
	25 yrs	18,098,855 (20,258,400)	n.r.	311	n.r.	58,196 (65,140)	4,365,018 (4,885,849)	n.r.	45	n.r.	98,538 (110,295)
	20 yrs	19,802,276 (22,165,073)	n.r.	313	n.r.	63,266 (70,815)	Ext dom	n.r.	Ext dom	n.r.	Ext dom
	*Universal genetic testing at different ages*										
	40 yrs	206,433,407 (231,064,928)	n.r.	546	n.r.	378,083 (423,196)	Ext dom	n.r.	Ext dom	n.r.	Ext dom
	35 yrs	241,672,941 (270,509,225)	n.r.	675	n.r.	358,034 (400,754)	Ext dom	n.r.	Ext dom	n.r.	Ext dom
	30 yrs	286,068,367 (320,201,888)	n.r.	800	n.r.	357,585 (400,252)	Ext dom	n.r.	Ext dom	n.r.	Ext dom
	25 yrs	338,661,509 (379,070,416)	n.r.	925	n.r.	366,121 (409,806)	320,562,655 (358,812,016)	n.r.	614	n.r.	522,008 (584,294)
	20 yrs	398,387,730 (445,923,136)	n.r.	933	n.r.	426,996 (477,945)	59,726,220 (66,852,720)	n.r.	8	n.r.	7,461,915 (8,352,267)

Dom: dominated, EB: endometrial biopsy, Ext dom: extended dominance, HBS: hypothetical biomarker panel screening including prolactin (sensitivity and specificity of 0.98, both), ICER: incremental cost-effectiveness ratio, ICUR: incremental cost-utility ratio, LYG: life years gained, OCP: oral contraceptive pills, QALY: quality-adjusted life year, TVS: transvaginal sonography, US: ultrasound, yrs: years. ^a^ calculated versus next (non-dominated) strategy; ^b^ risk of carrying a mismatch repair gene mutation associated with Lynch syndrome as measured by PREMM1, 2, 6 [22]; ^c^ mutation carriers were screened with transvaginal sonography and endometrial biopsy (and colonoscopy to early detect colorectal cancer), and were offered prophylactic procedures such as total abdominal hysterectomy and PBSO (and polypectomy to prevent colorectal cancer). *Italic: different risk levels of having the mutation.*

## Supplementary Materials

The following are available online at https://www.mdpi.com/2072-6694/12/7/1874/s1. Table S1. Search strategies. Databases searched and search codes; Table S2. List of abbreviations.
