# Supplementary material for: Cost-Effectiveness of Early Detection and Prevention Strategies for Endometrial Cancer—A Systematic Review
Source: Cancers (Basel). 2020 Jul 11;12(7):1874. doi: 10.3390/cancers12071874 (PMC7408795; doi:10.3390/cancers12071874)
Supplement: Supplementary file 1 [file cancers-12-01874-s001.pdf]

Supplementary Materials: Cost-Effectiveness of Early Detection and Prevention Strategies for Endometrial Cancer—A Systematic Review

Gaby Sroczynski, Artemisa Gogollari, Annette Conrads-Frank, Lára R. Hallsson, Nora Pashayan, Martin Widschwendter and Uwe Siebert

Table S1. Search strategies. Databases searched and search codes.

Database	Search Codes	Number of Publications Found
PubMed	Search strategy: 25/03/2019<br>Search (((((((endometr*) AND ((((neoplasm*) OR carcinom*) OR tumor*) OR cancer*))) OR "Endometrial Neoplasms"[Mesh])) AND (((detection) OR prevention) OR screening)) AND (((((economic*) AND *model*)) OR (((((Benefit*) OR Utilit*) OR Effectiv*)) AND cost*)) OR ((QALY) OR ((((qualit*) AND *adjusted*) AND life*) AND year*)))) AND (((((((((markov*) AND ((*model*) OR *chain*))) OR "Markov Chains"[Mesh]) OR "Monte Carlo Method"[Mesh]) OR ((decision*) AND (((*tree*) OR *analy*) OR *model*))) OR discrete event simulation) OR *simulat*) OR model*)	77 Hits
	Search update: 28/01/2020	0 Hits
CRD database	Search strategy: 28/04/2016<br>1. (Screening)<br>2. (Early detection)<br>3. (Prevention)<br>4. (endometr*) AND (cancer)<br>5. (microsimulation NEAR model*) OR (micro-simulation NEAR model*) OR (MISCAN)<br>6. (Markov NEXT model*) OR (mathematic* NEAR model*) OR (computer* NEAR model*)<br>7. (decision NEAR model*) OR (analytic* NEAR model*)<br>8. MeSH DESCRIPTOR Markov Chains EXPLODE ALL TREES<br>9. MeSH DESCRIPTOR Computer Simulation EXPLODE ALL TREES<br>10. #1 OR #2 OR #3<br>11. #5 OR #6 OR #7 OR #8 OR #9<br>12. #4 AND #10 AND #11	28 Hits
	Search update: 28/01/2020	0 Hits
Ovid Medline	Search strategy: 28/04/2016<br>1. (microsimulation adj3 model*).mp.<br>2. (analytic* adj5 model*).mp<br>3. (decision adj5 model*).mp<br>4. (computer* adj5 model*).mp<br>5. (Markov adj Model*).mp<br>6. (mathematic* adj model*).mp<br>7. (micro-simulation adj3 model*).mp<br>8. MISCAN.mp<br>9. exp Markov Chains<br>10. Prevention.ti,ab<br>11. Screening.ti,ab<br>12. Early detection.ti,ab<br>13. Endometrial Cancer.ti,ab<br>14. 10 or 11 or 12<br>15. 1 or 2 or 3 or 4 or 5 or 6 or 7 or 8 or 9<br>16. 13 and 14 and 15	9 Hits

	Search update: 28/01/2020	5 Hits
Embase	Search strategy: 28.04.2016<br>1. Endometrial Cancer.ti,ab<br>2. Early detection.ti,ab<br>3. Screening.ti,ab<br>4. Prevention.ti,ab<br>5. exp Computer Simulation<br>6. (microsimulation adj3 model*).mp<br>7. MISCAN.mp<br>8. exp Markov Chains<br>9. (micro-simulation adj3 model*).mp<br>10. (mathematic* adj model*).mp<br>11. (Markov adj Model*).mp<br>12. (decision adj5 model*).mp<br>13. (computer* adj5 model*).mp<br>14. 2 or 3 or 4<br>15. 5 or 6 or 7 or 8 or 9 or 10 or 11 or 12<br>16. 1 and 14 and 15	100 Hits
	Search update: 28/01/2020	11 Hits
EconLit	Search strategy: 28.04.2016<br>S1: Endometrial AND Cancer	1 Hit
	Search update: 28/01/2020	2 Hits
The Cochrane Library	Search strategy: 29.04.2016<br>1. Endometrial and Cancer (Word variations have been searched)<br>2. Screening or Prevention or Prophylactic or Early detection (Word variations have been searched)<br>3. microsimulation near model* (Word variations have been searched)<br>4. micro-simulation near model*:ti,ab,kw (Word variations have been searched)<br>5. MISCAN (Word variations have been searched)<br>6. Markov next Model* (Word variations have been searched)<br>7. MeSH descriptor: [Markov Chains] explode all trees<br>8. mathematic* near model* (Word variations have been searched)<br>9. MeSH descriptor: [Computer Simulation] explode all trees<br>10. computer* near model* (Word variations have been searched)<br>11. analytic* near model* (Word variations have been searched)<br>12. decision near model* (Word variations have been searched)<br>13. #3 or #4 or #5 or #6 or #7 or #8 or #9 or #10 or #11 or #12<br>14. #1 and #2<br>15. #13 and #14 in Economic Evaluations	2 Hits
	Search update: 28/01/2020	Hits
	Total	217 Hits
	Total after search update	235 Hits
	Total after removing the duplicates	125 Hits

Table S2. List of abbreviations.

**GDP-PPP**	**Gross Domestic Product - Purchasing Power Parities**
CPI	Consumer Price Index
LY	Life years
LYG	Life-years gained
QALY	quality-adjusted life years
ICER	Incremental cost-effectiveness ratio
ICUR	Incremental cost-utility ratio
Incr.	Incremental
CHEERS	The Consolidated Health Economic Evaluation Reporting Standards
WTP	Willingness-to-pay
WHO	World Health Organization

BMI	Body mass index
ACER	Average cost-effectiveness ratio,
PBSO	Prophylactic bilateral salpingo-oophorectomy
EB	Endometrial biopsy
yrs	Years
Dom	Dominated
n.r.	Not reported
Ext dom	Extended dominance
SA	Sensitivity analyses
TVS	Transvaginal sonography
CA-125	The cancer-antigen-125

© 2020 by the authors. Licensee MDPI, Basel, Switzerland. This article is an open access article distributed under the terms and conditions of the Creative Commons Attribution (CC BY) license (http://creativecommons.org/licenses/by/4.0/).
